# Flexural Strength of Two Multilayered and Monochromatic High Yttria Containing Zirconia Materials Following Different Sintering Parameters

**DOI:** 10.1055/s-0043-1772569

**Published:** 2023-09-20

**Authors:** Niwut Juntavee, Apa Juntavee, Chutikarn Jaralpong

**Affiliations:** 1 Department of Prosthodontics, Faculty of Dentistry, Khon Kaen University, Khon Kaen, Thailand; 2 Division of Pediatric Dentistry, Department of Preventive Dentistry, Faculty of Dentistry, Khon Kaen University, Khon Kaen, Thailand; 3 Division of Biomaterials and Prosthodontics Research, Faculty of Dentistry, Khon Kaen University, Khon Kaen, Thailand

**Keywords:** flexural strength, monolithic zirconia, multilayer zirconia, sintering temperature, sintering time

## Abstract

**Objectives** Sintering parameters influence the properties of zirconia. This study examined the effect of altering sintering temperature and time of monochrome and multilayer 5 mol% yttria-partially stabilized zirconia (5Y-PSZ) on flexural strength.

**Materials and Methods** Three hundred specimens (width × length × thickness = 10 × 20 × 2 mm) were prepared from monolayer (Z _X_ ) and multilayer (Z _M_ ) 5Y-PSZ and randomly sintered at decreasing (T _D_ : 1,450°C), regular (T _R_ : 1,500°C), and increasing (T _I_ : 1,550°C) sintering temperature, with extremely short (H _E_ : 10 minutes), ultrashort (H _U_ : 15 minutes), short (H _S_ : 30 minutes), and regular (H _R_ : 135 minutes) sintering time ( *n* = 15/group). The precrack was induced on the tension side before testing for flexural strength ( *σ* ).

**Statistical Analysis** Analysis of variance and Tukey's test were used for significant differences of *σ* at *p* < 0.05. The microstructure and crystalline (monoclinic; m, tetragonal; t, cubic; c) phase were evaluated by scanning electron microscope (SEM) and X-ray diffractometer (XRD).

**Results** Z _X_ T _I_ H _S_ indicated the highest *σ* for Z _X_ (315.81 ± 18.91 MPa), whereas Z _M_ T _I_ H _S_ indicated the highest *σ* for Z _M_ (335.21 ± 36.18 MPa). There was no significant difference for *σ* between Z _X_ and Z _M_ ( *p* > 0.05). Sintering zirconia at T _I_ or H _R_ indicated significantly higher *σ* than sintering at T _D_ or T _R_ or with H _S_ , H _E_ , or H _U_ for both Z _X_ and Z _M_ ( *p* < 0.05). There was no significant difference for *σ* between T _R_ H _R_ and T _I_ H _S_ , T _I_ H _U_ , and T _I_ H _E_ ( *p* > 0.05). SEM indicated intergranular and transgranular fractures. XRD revealed predominately c- and t-phases and minor amounts of m-phase.

**Conclusion** Increasing sintered temperature with decreasing time offers acceptable strength to regular sintering. Raising sintering temperature with decreasing time is suggested to facilitate chairside restorative reconstruction.

## Introduction

Dental ceramic restorations have been increasing as a result of their aesthetic properties, biological compatibility, and corrosion resistance. 1 Glass-reinforced ceramics are generally selected for aesthetic dental reconstruction owing to their translucence properties; nevertheless, they are of limited usage for extensive restorations because of their minimal fracture resistance and masking ability of the color from the underlining structures. Various novel dental ceramics have been established for improving mechanical properties to withstand the masticatory function for use as a long-span fixed partial denture. Zirconia ceramics possess many advantages, including their tooth-colored material, tremendous fracture strength, abrasion resistance, chemical durability, little thermal conduction, and high melting temperature. It has been increasingly used for many aspects of restorative dentistry, for example, a substructure for veneering ceramics, all-ceramic restoration, fixed partial prostheses, and full arch dental prostheses. 2 3 Zirconia is naturally occurring in pure forms of polymorphic crystalline structures. It possesses a monoclinic (m) phase at a temperature below 950°C, a tetragonal (t) phase at a temperature between 1,170 and 2,370°C, and a cubic (c) phase at a temperature above 2,370°C. Upon sintering of zirconia, a t-phase can be transformed to an m-phase at approximately 950°C. Vice versa, upon heating, an m-phase can be transformed into a t-phase at approximately 1,170°C. 2 A martensitic transformation property of zirconia can induce a strengthening phenomenon. 4 As a crack appears in zirconia, the tensile stress around the crack tip stimulates a t-phase to be transformed into an m-phase and leads to an increase in the volume of the crystalline by around 4 to 5%. 4 The volumetric enlargement causes compressive stress surrounding the crack tip to neutralize the further tensile stress, which can stop crack propagation. The transformation from the t→m phase improves the mechanical properties of the zirconia. Even though the t-phase is not a stable form at normal room temperature, adding soluble dopants, for example, yttrium oxide (Y _2_ O _3_ ), magnesium oxide (MgO), calcium oxide (Ca _2_ O _3_ ), cerium oxide (CeO _2_ ), and titanium oxide (Ti _2_ O _3_ ), is essential because they can enhance the stability of t-phase at normal room temperature. 5 The 3 mol% of Y _2_ O _3_ is a commonly used dopant for dental zirconia for forming yttria-stabilized tetragonal zirconia polycrystals (Y-TZP) that possesses remarkable strength over other ceramics. 6

The 3Y-TZP is dull white in color and quite opaque. It was primarily manufactured to produce substructures for porcelain veneering to achieve aesthetic translucence restoration. However, the problem with porcelain chipping is frequently reported. 1 6 This leads to the introduction of monolithic zirconia for the fabrication of dental restoration. 1 5 The monolithic zirconia can acceptably fabricate restoration at a reduced thickness (0.5–0.75 mm), compared with porcelain veneering restoration or other all-ceramics. 7 The conventional first-generation monolithic 3Y-TZP is still limited in translucency. Increasing the sintering temperature of conventional monolithic zirconia was able to induce a larger grain size, allowing more light transmission, thus better enhancing translucency, but decreasing the strength. 8 9 10 11 12 For improving the translucence of the second-generation monolithic zirconia, the quantity and particle size of the aluminum oxide (Al _2_ O _3_ ) were reduced. Upon the sintering process, the Al _2_ O _3_ particles were rearranged and settled at the grain boundaries. This resulted in less light scattering with Al _2_ O _3_ particles, promoting more light transmission through the zirconia grain, and providing better translucency, but still less translucency than glass-reinforced ceramics. Recently, a high percentage of Y _2_ O _3_ containing monolithic zirconia was introduced as 5 mol% (9.3 wt%) yttria partially stabilized zirconia (5Y-PSZ), that increased the c-phase up to 40 to 50% to replace the t-phase to an extent in translucent zirconia. 5 13 The c-phase occupied a greater volume and appeared farther isotropic than the t-phase, which exhibited less optical scattering effect from the grain boundary and provided more translucence. Recent monolithic zirconia was introduced in the form of multilayer zirconia that simulated the color of the natural tooth along the incisal to cervical regions, while monochrome zirconia contains only one color. 5

Zirconia dental restorations can be fabricated by milling the partially sintered block and further sintered at a temperature of 1,400°C for approximately 7 to 12 hours to derive fully sintered zirconia which needs designing compensation of approximately 20 to 25% for sintering shrinkage. 14 The sintering process is considered one of the utmost important stages in the fabrication of zirconia restorations in which they might be modified to optimize the properties of zirconia. A time-consuming sintering process is not suitable for the construction of zirconia restoration as one appointment (chairside) restorative treatment. Clinicians always look for a zirconia material which needs a short time to sinter to gain benefit from higher productivity and save energy and time. 15 Sintering parameters, such as the sintering temperature, sintered holding time, and heating rate, are frequently modified to obtain better properties of the zirconia restoration.

Strength is a crucial factor for the prediction of the performance of ceramic restorations. Fracture strength is usually used to evaluate the endurance of ceramic material. Ceramics with high endurance tend to be less susceptible to fractures. The strength of zirconia restorations is impacted by altering sintering parameters, such as sintered temperature and sintered time. It has been reported that increasing the sintered temperature or the sintered time can provide superior translucency of zirconia. 16 Contrarily, an extremely elevated sintered temperature was reported to reduce the fracture strength owing to the relocation of the Y _2_ O _3_ to the zirconia grain boundary. 9 Moreover, altering sintered parameters might disturb the character of the grain. With a large grain size, zirconia demonstrated a spontaneous transformation from t→m-phase, which may abbreviate its strength. The effect of altering the sintered parameter on the strength of zirconia is still controversial. Previous studies report that raising the sintered temperature and time for zirconia does not impact the hardness and flexural strength. 8 The sintering temperature of zirconia to be increased above 1,300°C results in an increased zirconia grain size. 8 Altering sintering temperature seems to indicate a significant negative correlation with flexural strength. 9 17 In contrast, the studies report that increasing sintered temperature in combination with reducing sintered time enables enhancing the flexural strength of zirconia. 18 19 Another study reports that sintering at a high sintering temperature with an increased sintering time provides more strength. 20 There are a few studies that assessed the consequence of short sintered times on the flexural strength of zirconia. 19 21 22 23

An adjustment of sintering parameters is an issue of interest due to the possibility of providing good mechanical properties of zirconia, saving processing time, and decreasing patient appointments. Most studies investigated the effect of sintered parameters on the strength of monochrome monolithic zirconia. 19 21 22 23 Up to this point, no study investigated the influence of altering the sintering parameters of yttrium oxide-contained monochrome and multilayer partially stabilized monolithic zirconia. Accordingly, this study aimed to evaluate the flexural strength of monochrome and multilayer monolithic 5Y-PSZ upon various sintered temperatures and sintered times. The hypothesis was that varying the sintered temperature and sintering time did not influence the flexural strength of monochrome and multilayer monolithic 5Y-PSZ.

## Materials and Methods

The sample size for this *in vitro* study was anticipated by the G*power 3.1 software (Heinrich-Heine-Universität, Düsseldorf, Germany) based on the statistical data in Yilmaz et al 24 using powers of test = 0.9, and *α* error = 0.05 as shown in Eq. (1) .



where: Z _α_ = standard normal deviation = 1.96 (α error = 0.05), Z _β_ = standard normal deviation = 1.28 (β error = 0.1), µ _1_ –µ _2_ = mean difference between experimental group (µ _1_ = 1040, µ _2_ = 931), and *σ* = standard deviation (SD) (σ _1_ = 121.33, σ _2_ = 31.66). The number of sample sizes based on this calculation was 15 specimens per group for this experiment.

### Preparation Zirconia Specimens

The 5Y-PSZ monochrome (Z _X_ : Cercon xt, Dentsply Sirona, Charlotte, North Carolina, United States) and multilayer (Z _M_ : Cercon xt ML, Dentsply Sirona) blocks were selected for this study, as shown in Table 1 . The zirconia specimens were initially prepared from presintered blocks, shade A2 of Z _X_ and Z _M_ (150 specimens each) as an enlarged dimension (width × length × thickness = 12.5 × 25 × 2.5 mm) to compensate for sintering shrinkage. A diamond-coated wheel (Mecatome T180, PRESI France, Eybens, France) was used to cut the specimens, which were then ground with waterproof SiC abrasive paper up to #1200 grit size and subsequently polished with diamond suspension (1 μm) in a polisher (Ecomet3, Beuhler, Lake Bluff, Illinois, United States). The samples were decontaminated for 10 minutes in an ultrasonic cleaner (Vitasonic II, Vita Zahnfabrik, Bad Säckingen, Germany) and finally dried for 60 minutes at room temperature. Each type of zirconia specimen was simply randomly allocated into 10 groups ( *n* = 15) under four different sintering times (extremely short [H _E_ : 10 minutes], ultrashort [H _U_ : 15 minutes], short [H _S_ : 30 minutes], and regular [H _R_ : 135 minutes]), and three different sintering temperatures (decreasing [T _D_ : 1,450°C], regular [T _R_ : 1,500°C], and increasing [T _I_ : 1,550°C]), as shown in Table 1 . The sintering process was performed using a ceramic furnace (inFire HTC, Dentsply Sirona) for both types of materials involved, heating at a rate of 22°C/min until reaching the temperature of 880°C and then continuing to heat at 11°C/min until the sintering temperature was reached, followed by cooling down to room temperature at a cooling speed of 35°C/min. Once the sintering process was completed, the specimen was digitally measured with a measuring device (Mitutoyo, Tokyo, Japan) for final dimension (width × length × thickness = 10 × 20 × 2 mm) and stored at room temperature for 24 hours before testing.

**Table 1:** Material, abbreviation (Abv), brand, and manufacturers, composition (wt%) of monochrome (Z _X_ ) and multilayer (Z _M_ ) 5 mol% yttria-partially stabilized zirconia (5Y-PSZ) sintered at decreasing (T _D_ ), regular (T _R_ ), and increasing (T _I_ ) sintering temperature (°C) with regular (H _R_ ), short (H _S_ ), ultrashort (H _U_ ), and extremely short (H _E_ ) sintering time (minute)

Material (Abv)	Brand and manufacturer	Composition	Sintering protocol				
			Temperature	Time			
Monochrome 5Y-TZP, (Z _X_ )	Cercon xt, Dentsply Sirona, Charlotte, NC, USA	≥ 99% ZrO _2_ + HfO _2_ + Y _2_ O _3_ , 9% Y _2_ O _3_ , < 3% HfO _2_ < 1% Al _2_ O _3_ + SiO _2_	1,450 (T _D_ )	10 (H _E_ )	15 (H _U_ )	30 (H _S_ )	
			1,500 (T _R_ )	10 (H _E_ )	15 (H _U_ )	30 (H _S_ )	135 (H _R_ )
			1,550 (T _I_ )	10 (H _E_ )	15 (H _U_ )	30 (H _S_ )	
Multilayer 5Y-TZP, (Z _M_ )	Cercon xt ML, Dentsply Sirona, Charlotte, NC, USA	≥ 99% ZrO _2_ + HfO _2_ + Y _2_ O _3_ , 9% Y _2_ O _3_ , < 3% HfO _2_ < 1% Al _2_ O _3_ + SiO _2_	1,450 (T _D_ )	10 (H _E_ )	15 (H _U_ )	30 (H _S_ )	
			1,500 (T _R_ )	10 (H _E_ )	15 (H _U_ )	30 (H _S_ )	135 (H _R_ )
			1,550 (T _I_ )	10 (H _E_ )	15 (H _U_ )	30 (H _S_ )	

### Evaluation Flexural Strength

**Fig. 1:**
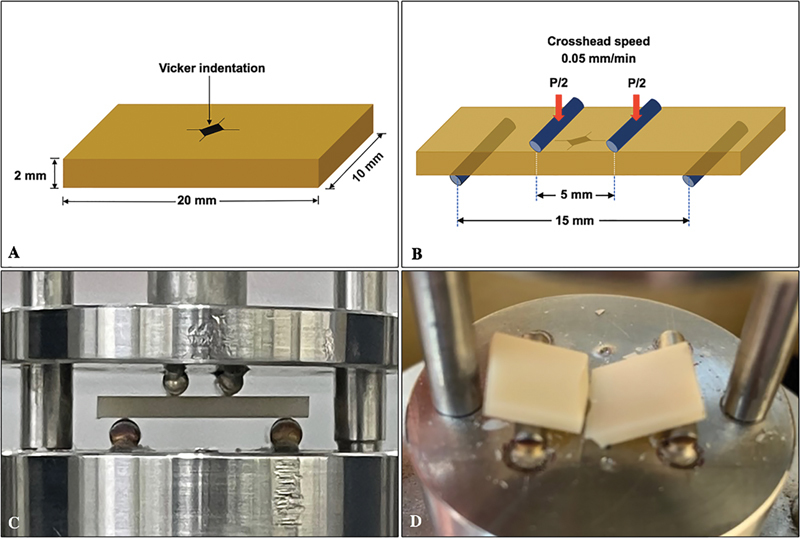
A rectangular ceramic specimen was precisely indented with Vickers indenter ( **A** ), placed on four-point bending test apparatus with Vickers indentation on the tension side ( **B** ), and loaded in a universal testing machine ( **C** ) until the ceramic specimen fractured ( **D** ).



The zirconia specimen was determined for flexure strength with a four-point flexure test. A precrack was initiated at the center of the sample using a Vickers microhardness diamond indenter (Future-tech FM800, Tokyo, Japan) at 1,000 g-force, loaded for 25 seconds indenting time to create a controlled precrack ( Fig. 1A ). 25 The indented specimen was placed in the four-point flexure test apparatus with the indented surface on the tension side, having an outer span of 15 mm and an inner span of 5 mm ( Fig. 1B ). A specimen was compressively loaded at a cross-head speed of 0.5 mm/min in a universal test machine (Lloyd, Leicester, United Kingdom) ( Fig. 1C ) until fracture ( Fig. 1D ). The fracture load (P; Newton) was verified and used for the calculation of flexural strength ( *σ* ; MPa) according to Eq. (2) .

where: *L* = length of the outer span (mm), *D* = specimen thickness (mm), and *W* = specimen width (mm)

### Determination of the Microstructure

The specimens were simply randomly chosen from each group for microscopic examination on the fracture surface. The specimen was cleaned with distilled water, dried with acetone, and then coated with gold-palladium in a sputtering machine (Emitech K-500X, Asford, United Kingdom) under vacuum 130 mTorr, at 10 mA current, for 180 seconds. The fracture surfaces of specimens were examined with a scanning electron microscope (SEM; Hitachi, Osaka, Japan) and energy dispersive spectroscopy (Oxford, Oxfordshire, United Kingdom) to characterize fracture surface and crystalline structure.

### Determination of the Phase Composition

The percentage of crystalline structures of zirconia specimens was measured using an X-ray diffractometer (XRD; PANalytical, Empyrean, Almelo, Netherlands). The copper k-α radiation was used to scan the specimens at 2-second intervals with a 0.02-step size at diffraction angles (2θ) of 25 to 55 degrees. The zirconia phase was evaluated by cross-referencing against the standard database from the Joint Committee of Powder Diffraction (PDF). The X'Pert Plus software (Philips, Almelo, Netherlands) was used to analyze the relative proportions of phases based on the peak intensity. The peaks were referenced based on PDFs No. 37–1484, 49–1642, and 42–1164, for the m-, t-, and c-phases, respectively. The proportion of the m-phase (X _m_ ) was estimated using the Garvie and Nicholson formulation as in Eq. (3) . The *I*_c_ , *I*_t_ , and *I*_m_ displayed the integrated intensities for the c-, t-, and m-phases, respectively. These were calculated by fitting a pseudo-Voigt distribution to the complimentary peaks and analyzing the area under the curves. To take the impact of yttria doping on the lattice parameters into consideration, a correction factor of 1.311 was determined using the nonlinear calibration curve of integrated intensity ratios versus volume fraction. The proportion of tetragonal (X _t_ ) and cubic (X _c_ ) phases was calculated as shown in Eqs. (4) and (5) . 26







### Statistical Analysis

An analysis of variance (ANOVA) and Tukey's honestly significant difference multiple comparisons was performed to justify significant differences in flexural strength of Z _X_ and Z _M_ subjected to different sintering times and sintering temperatures (α = 0.05) using statistics software (SPSS V-20, Chicago, Illinois, United States). The reliability of flexural strength was analyzed using Weibull statistics (ReliaSoft; Tucson, Arizona, United States) and determined for characteristic strength ( *σ _o_* ) and Weibull modulus ( *m* ) using Eq. (6) in concurrence with the slope of graph sketched between ln{ln(1/ *P*_s_ ( *V*_o_ ))} and *m* ln( *σ* / *σ _o_* ).



where: *P _s_* ( *V _o_* )is the survival probability of the sample, *σ* is the flexural strength, *σ _o_* is the characteristic strength, and *m* is the Weibull modulus.

## Results

**Fig. 2:**
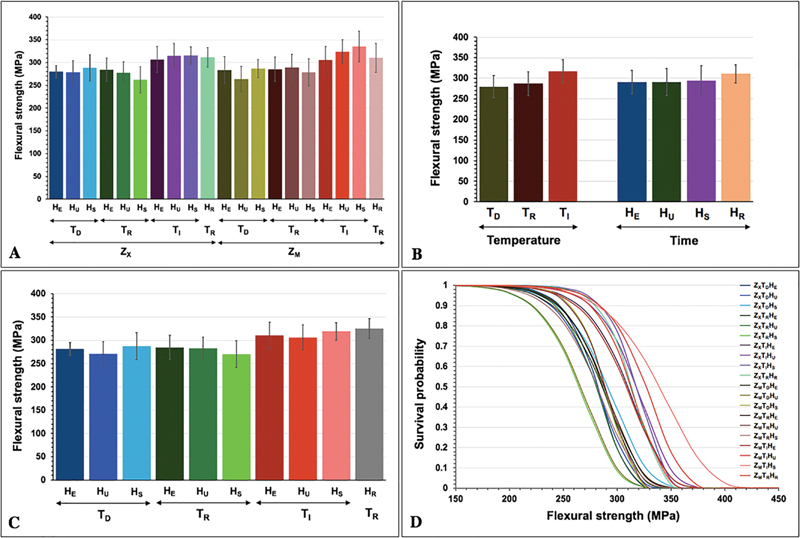
Flexural strength ( **A** – **C** ) and Weibull analysis ( **C** ) of 5Y-TZP of monochrome (Z _X_ ), and multilayer (Z _M_ ) 5Y-PSZ upon sintered at decreasing (T _D_ ), regular (T _R_ ), and increasing (T _I_ ) sintering temperature with regular (H _R_ ), short (H _S_ ), ultrashort (H _U_ ), and extremely short (H _E_ ) sintering time.

**Fig. 3:**
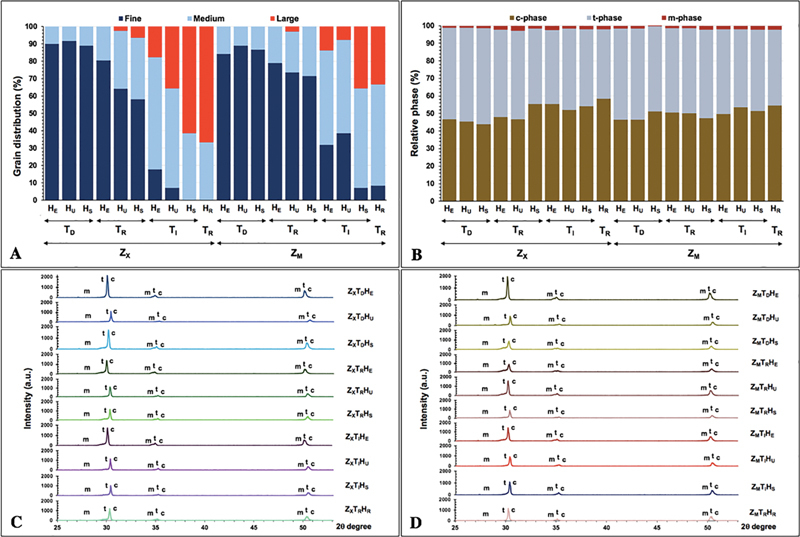
Grain distribution ( **A** ), relative phase composition ( **B** ), and X-ray diffraction pattern of monochrome (Z _X_ , **C** ) and multilayer (Z _M_ , **D** ) 5Y-PSZ upon sintered at decreasing (T _D_ ), regular (T _R_ ), and increasing (T _I_ ) sintering temperature with regular (H _R_ ), short (H _S_ ), ultrashort (H _U_ ), and extremely short (H _E_ ) sintering time.

The mean and SD of flexural strength ( *σ* ), 95% confidence interval (CI), Weibull modulus ( *m* ), and characteristic strength ( *σ _o_* ) of high yttrium oxide-contained monochrome and multilayer fully stabilized zirconia were presented in Table 2 and Fig. 2 . ANOVA signified that the flexural strength of 5Y-PSZ was significantly influenced by sintered time, sintered temperature, and the interaction between sintered time and sintered temperature of the sintering processes ( *p* < 0.05) but was not exaggerated by the category of monolithic zirconia ( *p* = 0.369), as shown in Table 3 . The mean flexural strength for the multilayer zirconia (295.8 ± 33.75 MPa) was not significantly different compared with the monochrome zirconia (291.84 ± 30.37 MPa) ( *p* = 0.369). In the monochrome zirconia group, the Z _X_ T _I_ H _S_ group showed the highest flexural strength (315.81 ± 18.91 MPa), while the Z _X_ T _R_ H _S_ group showed the lowest flexural strength (261.9 ± 28.59 MPa) ( Fig. 2A ). In the multilayer zirconia group, the Z _M_ T _I_ H _S_ group presented with maximum flexural strength (335.21 ± 33.85 MPa), while the Z _M_ T _D_ H _U_ group presented with minimum flexural strength (263.29 ± 27.92 MPa) ( Fig. 2A ). This study indicated that types of zirconia have no significant effect on the flexural strength ( *p* > 0.05), whereas altering either the sintering temperature or sintering time has a significant influence on the flexural strength of either the monochrome or multilayer zirconia ( *p* < 0.05). Multiple comparisons between the groups of monolithic zirconia were presented in Table 4 . The study revealed that the sintering process of 5Y-TZP at an increasing sintered temperature (T _I_ , 1,550°C) produced a significantly greater flexural strength than sintering at a regular sintered temperature (T _R_ , 1,500°C) ( *p* < 0.001) for both monochrome and multilayer zirconia. Sintering 5Y-TZP at a decreasing sintered temperature (T _D_ , 1,450°C) produced a significant reduction in flexural strength compared with sintering at a regular sintered temperature (T _R_ , 1,500°C) ( *p* < 0.001) for both monochrome and multilayer zirconia, as shown in Table 4 and Fig. 3B . The study indicated that sintering zirconia for a shorter sintering time generated a significantly lesser flexure strength than sintering at a regular sintered time ( *p* < 0.001) for both monochrome and multilayer zirconia, as presented in Table 4 and Fig. 2B . The post hoc Tukey's comparisons suggested that sintering 5Y-TZP, either Z _X_ or Z _M_ , at a decreasing sintering temperature, combined with a reduced sintering time, resulted in a significant depreciation in flexural strength ( *p* < 0.001), compared with sintering at a regular sintering temperature and time (T _R_ H _R_ ), as presented in Table 4 and Fig. 2C . The multiple comparisons indicated that sintering zirconia, either Z _X_ or Z _M_ , with short (H _S_ ), ultrashort (H _U_ ), or extremely short (H _E_ ) sintering times and an increased sintering temperature (T _I_ ) resulted in no significant difference in flexural strength ( *p* > 0.05), compared with sintering at a regular sintering temperature and time (T _R_ H _R_ ), as presented in Table 4 and Fig. 2C . The study indicated no significant differences in the flexural strength among groups sintered at a decreasing sintering temperature with short (H _S_ ), ultrashort (H _U_ ), or extremely short (H _E_ ) sintering times ( *p* > 0.05). The result also revealed no significant differences in flexural strength among the groups sintered at an increasing sintering temperature (T _I_ ) with regular (H _R_ ), short (H _S_ ), ultrashort (H _U_ ), or extremely short (H _E_ ) sintering times ( *p* > 0.05), as presented in Table 4 and Fig. 2C . The characteristic strength ( *σ _o_* ) and Weibull modulus ( *m* ) for each group of zirconia are presented in Table 2 . The *m* for the tested monolithic zirconia ranged from 10.15 to 16.50. The Z _X_ T _I_ H _S_ group indicated the highest *m* (16.50), while the Z _M_ T _D_ H _U_ group indicated the lowest *m* (10.15). The survival function graphs of the monochrome and multilayer zirconia are shown in Fig. 2D .

**Table 2:** Mean, standard deviation (SD) of flexural strength (MPa), characteristic strength ( *σ*_0_ ), Weibull modulus ( *m* ), relative cubic (c-), tetragonal (t-), and monolithic (m-) phase content (wt%), and grain size distribution (%) of monochrome (M), and multilayer 5Y-PSZ upon sintered at decreasing (T _D_ ), regular (T _R_ ), and increasing (T _I_ ) sintering temperature with regular (H _R_ ), short (H _S_ ), ultrashort (H _U_ ), and extremely short (H _E_ ) sintering time

Group	*σ* (MPa)	*σ*_o_ (MPa)	*m*	Relative phase (wt%)			Grain size distribution (%)		
	Mean ± SD			c-	t-	m-	Small	Medium	Large
Z _X_ T _D_ H _E_	279.35 ± 21.55	290	13.85	46.7	52.2	1.2	90	10	0
Z _X_ T _D_ H _U_	278.20 ± 26.04	290	11.09	44.5	53.3	1.2	91.67	8.33	0
Z _X_ T _D_ H _S_	288.21 ± 28.80	302	10.59	43	54.7	1.3	88.89	11.11	0
Z _X_ T _R_ H _E_	284.11 ± 25.70	296	12.20	47.9	50	2.1	80.49	19.51	0
Z _X_ T _R_ H _U_	277.64 ± 24.18	289	11.44	46.7	50.5	2.9	64.10	33.33	2.56
Z _X_ T _R_ H _S_	261.90 ± 28.59	274	10.18	55.5	43.1	1.5	58.06	35.48	6.45
Z _X_ T _I_ H _E_	306.80 ± 28.08	320	10.79	55.4	42	2.5	17.65	64.71	17.65
Z _X_ T _I_ H _U_	315.06 ± 26.87	327	13.02	52.1	46.3	1.6	7.14	57.14	35.71
Z _X_ T _I_ H _S_	315.80 ± 18.91	326	16.50	54.1	44	1.9	0	38.46	61.54
Z _X_ T _R_ H _R_	311.35 ± 21.29	321	16.42	58.6	39.5	2	0	33.33	66.67
Z _M_ T _D_ H _E_	283.16 ± 29.46	296	10.59	46.4	52	1.6	84.09	15.91	0
Z _M_ T _D_ H _U_	263.29 ± 27.92	276	10.15	46.5	52	1.5	88.99	11.11	0
Z _M_ T _D_ H _S_	286.65 ± 20.13	296	14.67	51.1	48.7	0.3	86.67	13.33	0
Z _M_ T _R_ H _E_	285.37 ± 26.76	298	11.47	50.5	48.1	1.4	78.95	21.05	0
Z _M_ T _R_ H _U_	287.03 ± 21.50	297	14.79	50.1	48.4	1.4	73.53	23.53	2.94
Z _M_ T _R_ H _S_	278.29 ± 29.85	292	10.32	47.3	50.5	2.1	71.43	28.53	0
Z _M_ T _I_ H _E_	305.36 ± 29.65	336	11.45	49.6	48.5	1.9	31.82	55.55	13.64
Z _M_ T _I_ H _U_	323.74 ± 26.51	336	13.36	53.5	44.5	2	38.46	53.85	7.69
Z _M_ T _I_ H _S_	335.21 ± 33.85	352	10.42	51.4	46.3	2.2	7.14	57.14	35.71
Z _M_ T _R_ H _R_	310.75 ± 21.86	321	14.39	54.6	43.1	2.3	8.33	58.33	33.33

**Table 3:** ANOVA of flexural strength of monochrome and multilayer 5Y-PSZ upon sintered at decreasing (T _D_ ), regular (T _R_ ), and increasing (T _I_ ) sintering temperature with regular (H _R_ ), short (H _S_ ), ultrashort (H _U_ ), and extremely short (H _E_ ) sintering time

Source	SS	df	MS	*F*	*p* -Value
Corrected model	116,017.78	19	6,106.199	8.887	0.001
Intercept	24,223,174.8	1	24,223,174.8	35,254.252	0.001
Material	622.072	1	622.072	0.905	0.342
Time	23,377.835	3	7,792.612	11.341	0.001
Temp	84,683.761	2	42,341.881	61.624	0.001
Material*Time	2,172.332	3	722.11	1.042	0.369
Material*Temp	2,599.093	2	1,299.546	1.869	0.153
Time*Temp	13,125.892	4	3,281.473	4.72	0.001
Material*Time*Temp	2,297.348	4	574.337	0.836	0.503
Error	182,387.827	280	687.099		
Total	2,610,046.9	300			
Corrected total	208,405.607	299			

Abbreviations: ANOVA, analysis of variance; df, degree of freedom; *F* , *F* -ratio; MS, mean square; SS, sum of squares.

**Table 4:** Post hoc Tukey's multiple comparisons (A) of flexural strength of monochrome (Mo) and multilayer (Mu) 5Y-PSZ upon sintered at decreasing (T _D_ ), regular (T _R_ ), and increasing (T _I_ ) sintering temperature with regular (H _R_ ), short (H _S_ ), ultrashort (H _U_ ), and extremely short (H _E_ ) sintering time

**(A) Post hoc of flexural strength as a function of either sintering temperature or sintering time**										
	** T _D_**	** T _R_**	** T _I_**				** H _E_**	** H _U_**	** H _S_**	** H _R_**
** T _D_**	1	0.127	0.001			** H _E_**	1	1	0.786	0.002
** T _R_**		1	0.001			** H _U_**		1	0.804	0.002
** T _I_**			1			** H _S_**			1	0.017
						** H _R_**				1
**(B) Post hoc of flexural strength as a function of sintering temperature and sintering time**										
**Factors**	** T _D_ H _E_**	** T _D_ H _U_**	** T _D_ H _S_**	** T _R_ H _E_**	** T _R_ H _U_**	** T _R_ H _S_**	** T _I_ H _E_**	** T _I_ H _U_**	** T _I_ H _S_**	** T _R_ H _R_**
** T _D_ H _E_**	1	0.874	0.996	1	1	0.829	0.012	0.001	0.001	0.001
** T _D_ H _U_**		1	0.303	0.564	0.795	1	0.001	0.001	0.001	0.001
** T _D_ H _S_**			1	1	0.999	0.251	0.166	0.001	0.001	0.025
** T _R_ H _E_**				1	1	0.497	0.020	0.001	0.001	0.006
** T _R_ H _U_**					1	0.739	0.020	0.001	0.001	0.002
** T _R_ H _S_**						1	0.001	0.001	0.001	0.001
** T _I_ H _E_**							1	0.633	0.126	1
** T _I_ H _U_**								1	0.997	0.96
** T _I_ H _S_**									1	0.477
** T _R_ H _R_**										1

The microstructure observed in the grain size of the tested zirconia indicated the smallest grain size was 0.3 µm and the largest grain size was 2.1 µm. The grain sizes (µm) were defined for the zirconia crystal structures into three categories, which were: small (0.3–0.9 µm), medium (0.91–1.5 µm), and large (1.5–2.1 µm). The percentage (%) of the small, medium, and large grain sizes for each zirconia group is shown in Table 2 and Fig. 3A . The monochrome monolithic zirconia group (Z _X_ ) that was sintered at a regular sintered temperature (T _R_ ) and regular sintered time (H _R_ ) revealed crystal structures that were primarily composed of large grains and a minor amount of medium grains. The multilayer monolithic zirconia group (Z _M_ ) that was sintered at a regular sintered temperature (T _R_ ) and regular sintered time (H _R_ ) revealed crystal structures that were primarily composed of medium grains and a minor amount of large grains, as shown in Table 2 and Fig. 3A . The altering sintering parameters gave rise to the difference in grain sizes of zirconia, as presented in Table 2 and Fig. 3A . Decreasing the sintering temperature expedited the tiny grains while increasing the sintered temperature induced the growth of the zirconia grain. Increasing the sintering temperature induced a vaster number of large size of zirconia grains than sintering at regular or decreasing temperatures. The study indicated that the lengthier the sintered time, the increase in the amount of greater grain exhibited, as shown in Table 2 and Fig. 3A .

The microscopic analysis of phase distribution of the 5Y-PSZ using XRD disclosed that the position of a peak for the samples matched the m-, t-, and c-phases for ZrO _2_ . The concentrations concerning a weight percentage (wt%) of monoclinic (X _m_ ), tetragonal (X _t_ ), and cubic (X _c_ ) phases are illustrated in Fig. 3B and Table 2 . The XRD configurations discovered the principal crystal structure of the c- and t-phases with a minimal quantity of the m-phase for all tested groups. The intensity of the peak provided information about the quantity of the crystalline phase. The major peaks for t-phase were detected at the diffraction angle (2θ, degree) of 30.28 degrees which is associated with the 101-crystalline structure. The additional peaks of the t-phase were noticed at the 2θ of 35.271 and 52.28 degrees which are associated with the 002- and the 110-crystalline plane of the t-phase. Most of the peak intensity of the c-phases was found at the 2θ of 30.28 degrees associated with the 111-crystallographic plane, while the other peak intensity of the c-phase was found at the 2θ of 35.29 and 52.31 degrees. The most intensity of the peak for the m-phase was found at the 2θ of 28.48 degrees which matched the 111-crystallographic plane, while the lesser part of the intensity of the peak for the m-phase was found at the 2θ of 34.75 and 52.31 degrees, which matched to the 11ī- and 120-crystallographic plane. The Z _X_ T _R_ H _S_ provided the highest c-phase, Z _M_ T _D_ H _U_ provided the highest t-phase, and Z _M_ T _R_ H _S_ provided the highest m-phase. The Z _M_ T _D_ H _U_ provided the lowest c-phase, Z _M_ T _R_ H _E_ provided the lowest t-phase, and Z _X_ T _D_ H _E_ provided the lowest m-phase, as shown in Table 2 and Fig. 3C and D . The relative phase concentration (wt%) varied upon the type of zirconia, sintered temperature, and sintered time.

**Fig. 4:**
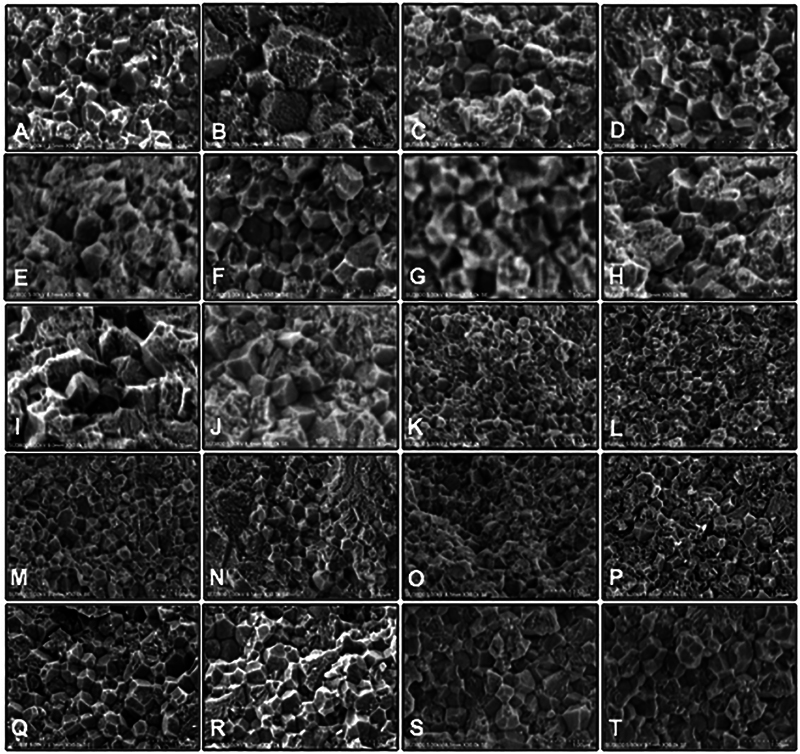
Scanning electron microscope photomicrographs of a crystalline structure indicated grain size and grain distribution at ×30K magnification of 5Y-PSZ monolayer ( **A** – **J** ) and multilayer ( **K** – **T** ) upon sintered at decreasing ( **A** , **B** , **C** , **K** , **L** , **M** ), regular ( **D** , **E** , **F** , **J** , **N** , **O** , **P** , **T** ), and increasing ( **G** , **H** , **I** , **Q** , **R** , **S** ) sintering temperature with regular ( **J** , **T** ), short ( **C** , **F** , **I** , **M** , **P** , **S** ), ultrashort ( **B** , **E** , **H** , **L** , **O** , **R** ), and extremely short ( **A** , **D** , **G** , **K** , **N** , **Q** ) sintering time.

The SEM was used to observe the fracture surface of the zirconia. The fracture indicated a crack origin at the Vickers indentation crack. The precrack acted as a stress concentration area in the tested specimen and created a crack tip on the tensile side. The crack propagated from the tensile side and quickly ran into the compressive zone until a catastrophic failure happened in the test specimen. The SEM image indicated that the crack propagated to the radial striation from the tensile side to the compressive side, as presented in Fig. 4 . Based on the crack patterns of brittle material, mist and hackle patterns were found in all groups. The hackle lines were not obvious in Z _X_ T _D_ H _S_ , Z _X_ T _R_ H _E_ , Z _M_ T _D_ H _S_ , and Z _M_ T _R_ H _U_ . The porosity was not found in all groups of material structure. The ultrastructural micrographs obtained from the fractography analysis are presented in Fig. 4 at ×30K magnification. The photomicrographs revealed that the grain size increased with the sintering temperature. Intergranular and transgranular fractures were found in all groups. The higher the sintering temperature was induced, the more transgranular fracture patterns appeared. Intergranular fractures occurred when the crack propagated along the grain boundaries of the material, while transgranular fractures occurred when the crack grew through the grains of the materials.

## Discussion

The strength of dental restoration is important for long-term success in clinical practice. This study examined the impact of varying sintering parameters on the flexure strength of monochrome and multilayer monolithic 5Y-PSZ. The result revealed that altering the sintering time and temperature affected flexural strength for both monochrome and multilayer monolithic zirconia. However, no significantly different flexure strength between monochrome and multilayer monolithic zirconia upon varying the sintering parameters was denoted. Thus, the null hypothesis was rejected for altering sintering time and temperature but accepted for types of monolithic zirconia. The study suggested that the flexure strength of 5Y-PSZ increased when the sintered temperature was increased. The study revealed that firing monolithic zirconia at an escalating temperature provided a greater flexural strength while decreasing the sintering temperature tended to reduce flexural strength. This is probably related to the increasing sintering temperature enabling the elimination of pores in the microstructure by fusing grain particles. The flexural strength increases when enhancing the sintering temperature because the particles of zirconia are efficiently jointing, and therefore, the pores at the grain boundary tend to diminish and increase the density of the material, which leads to raising the strength of the zirconia. 9 The study was in agreement with other studies that reported increasing flexural strength when the sintering temperature was increased. 8 However, the results of this study were not endorsed by other previous studies that reported decreasing flexural strength when the sintering temperature was increased. 9 17 The differences in the results are possibly related to the other studies varying the sintering temperature within a narrower range than this study, and the same goes for the difference in altering the sintering times and zirconia brands tested. Furthermore, this study demonstrated no significant difference in the flexure strength of 5Y-PSZ upon the regular sintering program (T _R_ H _R_ ) and increasing the sintering temperature with a short holding time for both monochrome and multilayer monolithic zirconia groups. This study demonstrated that raising the sintering temperature of monolithic zirconia and reducing the sintered holding time provided a suitable degree of densification, and grain developed completely comparable to regular sintering parameters. As the porosities were decreased, the bending strength of zirconia was increased. The results agreed with the former study, which suggested that sintering zirconia at 1,500 to 1,550°C would compromise mechanical properties and provide good optical properties. 9 This study revealed that sintering with a regular sintering program produced comparable flexure strength with sintering at increased temperatures and short holding times. This is probably related to how the sintering process at an exceeding temperature tended to reduce the number of pores as well as eliminated the minute pores, which were located between the grains. The pores are the causes of internal defects and increased inner stress that results in a reduction of the flexural strength of the material, as well as a reduction of the area of a solid-phase connection. 27 The sintering process at an increasing sintering temperature tended to coalesce many minute pores and formed a minimal amount of larger pores at the grain boundaries. It has been reported that decreased sintering temperatures provided a small grain size, resulting in a stronger material. This study exhibited that the flexural strength was reduced when the 5Y-PSZ was sintered at a decreased sintered temperature and time. Upon sintering at a reduced sintered temperature with a short holding time, the zirconia was likely not sintered quite densely, and therefore, easily fractured. 26

Altering the sintered time exaggerated the flexure strength of zirconia. Sintering zirconia with a regular sintered holding time produced a significantly different flexural strength with sintering at extremely short, ultrashort, and short holding times. Nevertheless, no significant difference in flexural strength was indicated when sintering at extremely short, ultrashort, and short holding times. This is probably related to the appropriate ability of crystallization and grain growth of zirconia established upon the reduction of the sintered holding time. Also, the possibility of minute pores being eliminated, as well as pores coalescing at the grain boundaries, was established upon a reduction of the sintered holding time. However, this weak point of crystallization of zirconia sintered at the short sintering time was compensated by increasing the sintering temperature. The study was confirmed by other previous studies. 18 21 However, the results of this study were preceded by a former study that reported the sintering time did not affect the strength of zirconia. 28 This is possibly associated with the differences in the designed studies regarding the sintering time to facilitate appropriate grain fusion and minute pore elimination. Moreover, the different results may be due to our narrow range of altering the sintering times with changing temperatures. These results exhibited no significant effect from the type of monolithic zirconia on flexural strength. This study revealed that pigments added into each layer of a multilayer monolithic zirconia group do not affect the strength of monolithic zirconia. This was supported by other studies. 29 30

The Weibull analysis was established on the theory of the failure of the material, and the distribution of strength was defined effectively in terms of the Weibull modulus ( *m* ). The *m* parameter described the statistical behavior of the strength and related to the distribution of physical flaws in the brittle material. In this study, the *m* ranging from 10.15 to 16.50 indicated that the *m* of all testing groups is reliable based on their compliance with the acceptable range for dental ceramics ( *m* = 5–15). The greater the *m* value of the material indicates, the more consistent the material possesses. It could be inferred that the material's physical defects, whether they come from the substance itself or the production process, are dispersed evenly throughout the specimen. 31

The fractographic analysis indicated that the crack originated from the indentation notch and propagated from the tensile side, quickly running into the compressive zone. Based on the fractography of brittle material, a hackle pattern was found in all groups. The hackle mark was an indication of the crack propagation direction. 32 This area was associated with a large amount of strain energy absorption. The fracture lines were not interrupted, but the fracture lines were not obvious in Z _X_ T _D_ H _S_ , Z _X_ T _R_ H _E_ , Z _M_ T _D_ H _S_ , and Z _M_ T _R_ H _U_ . The crack may easily cross the material and fracture. The obscure hackle line pattern was related to the low flexure strength of the zirconia tested. While the sintering temperature increased with the short sintering time group, the controlled group showed an obvious hackle line. This correlated with the results; both the increased sintered temperature and short sintered time group demonstrated high flexure strength. Porosity is one of the factors that can be related to flexural strength. 32 The porosity acts as a stress concentration area that can lead to the initiation and propagation of cracks, thus reducing the flexural strength of the material. 18 32 There were no pores present in the structure of monolithic zirconia. Moreover, the SEM photomicrograph of the crystalline structure at the fracture area showed that the sintering temperature affected the crystallographic structure. The grain size increased when the sintering temperature was enhanced. There were mixtures of intragranular and transgranular fracture patterns in all groups, and the transgranular fractures occurred when the crack grew through the material grains. As an analogy, in a wall of bricks, intergranular fractures would correspond to a fracture in the mortar keeping the bricks together. In a transgranular fracture pattern, the crack propagation would trail in a path where the utmost stress intensity is indicated under the loading situations. Intergranular fractures occur when grain boundaries are weak, resulting in decohesion at grain boundaries. Grain boundaries are regions with many faults, dislocations, and voids. Intergranular fractures generally increase by decreasing the grain size and increasing the grain boundaries, which lead to increased speed of crack growth and low flexural strength. 31 This correlates with the results: sintering the zirconia at a low temperature provided a small grain size and intergranular fracture area. Upon decreasing sintering temperatures, inadequate energy for grain consolidation caused weak grain boundaries. 27 32 Enhancing the sintering temperature with a short sintering time group and control group provides a more transgranular fracture mode. In general, the transgranular fracture energy has higher energy than intergranular fractures. 27 32 This correlates with the increased flexural strength in both sintering temperatures with short sintering times and control groups. Sintering at an excessive temperature for a short period may provide suitable densification, eliminate porosity, and complete grain growth, resulting in acceptable strength. 27 32

The crystalline consideration of the zirconia with XRD discovered a match between the peak position of the samples and the corresponding m-, t-, and c-phases for ZrO _2_ . The XRD patterns discovered the principal c- and t-phases with minimal amounts of the m-phase in 5Y-PSZ. Varying the sintering parameters affects the zirconia phase. Raising the sintered temperature and time tend to increase the c-phase. The c-phase concentration in the specimens was negligible in the concentration difference of the zirconia brands, which were 5Y-PSZ. The limitation of this study is that only one brand of 5Y-PSZ was examined. The results might not be extrapolated for 3Y-TZP and 4Y-TZP monolithic zirconia. Additionally, these results may be specific to the zirconia brand and the computer-aided design/computer-aided manufacturing system used. Furthermore, the specimens in this study were prepared in bars shape, which provides more benefits of uncomplex design for uniaxial flexural strength test and delivers more reliable results compared with anatomical shape specimens. Further studies should investigate other properties of the material such as optical properties when sintering parameters are altered.

## Conclusion

With the well-control experiment, it can be concluded that varying sintered temperature and sintered time affected the flexural strength of high yttrium oxide-contained monochrome and multilayer fully stabilized zirconia. Sintered zirconia at a raised sintered temperature can increase flexural strength, while reducing sintering temperature reduces flexural strength. Reducing the sintered holding temperature results in a reduction in flexural strength. Nevertheless, the sintered holding time can be reduced without jeopardizing the flexure strength of the 5Y-PSZ in the condition of sintering at a high temperature. The study recommends increasing the sintering temperature by approximately 50°C higher than the regular manufacturer's suggestion, in case speeding the sintering time is performed.

## Clinical Implication

This study suggested a fabrication method for the performance of monolithic zirconia restoration. The procedure could save time and ensure reliability from the sintering process. Sintering at an excessive temperature for a short sintering period offers acceptable flexural strength, and this strength is not different from that of a regular program. The conventional sintering parameters take a long time, so this is an alternative method that can provide effective time and good output. Technicians could apply sintering at a high temperature in a short time for convenient and productive manufacturing. Clinicians could also use this program for chairside applications in one-visit restorative practice with high yttrium oxide-doped monochrome and multilayer fully stabilized zirconia.
